# Prospective validation and implementation of a model to identify patients with carbapenem-resistant Enterobacterales (CRE) carriage on admission to acute care hospitals

**DOI:** 10.1017/ice.2026.10488

**Published:** 2026-06-16

**Authors:** Hyun Bin Kim, Radhika Prakash-Asrani, Chris W. Bower, Chad Robichaux, Barney Chan, Sarah W. Satola, Madeleine Boulis, Alexandra Rios, Twinkle Trehan, Kripa Michalin, Jesse T. Jacob, Scott K. Fridkin, Jessica Howard-Anderson

**Affiliations:** 1 Department of Medicine, Emory University School of Medicine, Atlanta, GA, USA; 2 Division of Infectious Diseases, Department of Medicine, Emory University School of Medicine, Atlanta, GA, USA; 3 Division of Biomedical Informatics, Department of Medicine, Emory University School of Medicine, Atlanta, GA, USA

## Abstract

**Objective:**

Use the electronic health record (EHR) to prospectively validate a risk prediction tool identifying patients at high-risk for carbapenem-resistant Enterobacterales (CRE).

**Design:**

Prospective, cross-sectional analysis.

**Participants:**

Adults admitted to two hospitals in Atlanta, Georgia.

**Methods:**

An EHR report calculated a CRE risk score on all admissions from 6/2024–3/2025. The risk score was determined from a prior model incorporating data from current and prior hospitalizations. Stool or perianal samples were cultured from a convenience sample of patients with the highest risk scores. A receiver operating curve analysis calculated sensitivity, specificity, positive predictive value (PPV), and negative predictive values (NPV) of various risk scores that could be used as a “threshold” for CRE admission testing. Using the threshold with the greatest Youden’s index, we estimated the cost of implementing a CRE screening program.

**Results:**

Of the 853 patients approached, 342 (40%) consented. Eleven (3.2%) tested positive for CRE. Patients with CRE had a higher median CRE risk score (0.19% vs 0.04%) than those who tested negative. The AUC of the model was 0.66. Using a testing threshold of 0.16% yielded a 55% sensitivity, 84% specificity, 10% PPV, and 98% NPV. The number needed to screen to diagnose 1 patient with CRE was 12 patients, and the screening program approximately costs $8,015/month.

**Conclusions:**

An EHR-based risk prediction tool can detect patients likely to be colonized with CRE. In facilities with a low CRE prevalence, identifying a high-risk subset of patients to test could be a cost-effective infection prevention initiative.

## Introduction

Carbapenem-resistant Enterobacterales (CRE) is an ongoing threat to public health and patient safety, now amplified in a recent report from the Centers for Disease Control and Prevention (CDC) demonstrating an increased incidence of carbapenemase-producing CRE infections over the past 4 years.^1–5^ Although some patients present acutely with infections caused by CRE, many patients with CRE have asymptomatic colonization that persist for long periods of time and later progress to clinical infection. A pooled meta-analysis shows that roughly one-third of those with CRE colonization in the gastrointestinal tract remain colonized one year after initial identification.^6^

Asymptomatic CRE carriers can contribute to patient-to-patient transmission of CRE in healthcare settings and healthcare-associated outbreaks.^7^ Although detection of CRE carriers and placement of these individuals in transmission-based precautions can limit transmission, testing all admitted patients for CRE is cost- and labor-prohibitive.^8,9^ Most US hospitals do not have a comprehensive program to identify CRE carriers, and since most CRE carriers admitted to hospitals lack clinical evidence of CRE, they are never placed in transmission-based precautions.^8,10,11^ An efficient and cost-effective process is needed to rapidly identify and test patients who are likely to be CRE carriers.

Administrative and clinical data available at the time of admission have previously been used to identify patients at highest risk for healthcare-acquired infections.^12–16^ Prior studies in intensive care units have also assessed surveillance testing for CRE.^17,18^ Our study adds to the existing literature by using a risk prediction model to stratify patients at higher risk for CRE and includes all adult inpatients. Our CRE risk prediction model was originally created using Chicago data and then modified and optimized for our own academic healthcare network in Atlanta, GA, and incorporated into an electronic health record (EHR) report.^14,15^ The model uses age, prior healthcare admissions, comorbidities, prior antibiotic days of therapy, and infection diagnosis in the last year to determine the probability of having CRE in a clinical culture within the first three hospital days (“CRE risk score”). We used the daily EHR report to identify patients likely to be CRE carriers and prospectively validated the model by testing a subset of those patients for CRE. We also explored which CRE risk score would be the most effective and cost-efficient for our hospitals to use as a threshold for testing for CRE on admission.

## Methods

### Setting and patient sampling

This study was conducted at a 605-bed community-academic, tertiary care hospital and a 751-bed academic, quaternary care hospital in the same healthcare network in Atlanta, GA from June 2024 through March 2025. Both hospitals do not routinely perform surveillance testing for CRE.

**Figure 1.**
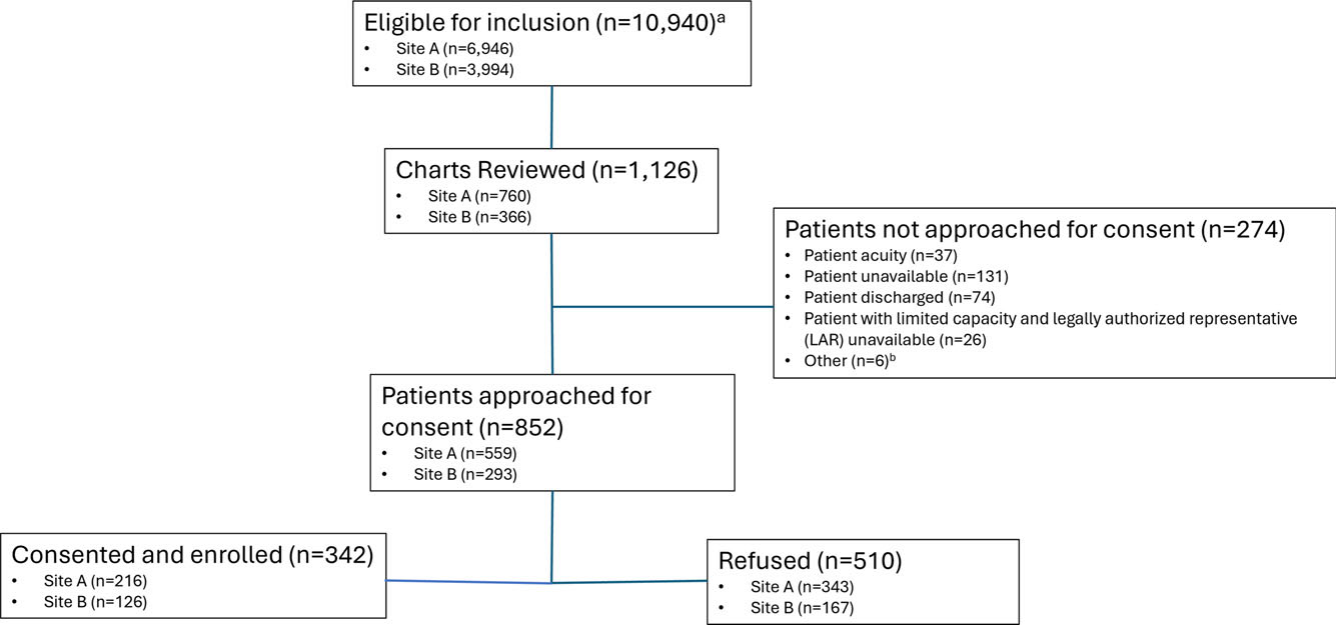
Figure 1 long description. Research staff reviewed a list of admitted patients from the prior calendar day and reviewed the electronic medical record of approximately 10–15 patients per day with the highest CRE risk scores and attempted to approach and consent these patients for study enrollment. (a) This does not include patients <18 years old and those admitted to labor and delivery/maternity and hospice units as they were excluded. (b) Other reasons included non-English speaking, patients with a colostomy (before change in protocol), enrolled in prior admission and prior refusal to participate.

Study staff reviewed an EHR report (Epic Reporting Workbench) of admitted patients from the prior calendar day on periodic sampling days (approximately 2 days/week, Monday–Friday). Using this report, staff then performed a chart review of approximately 10–15 eligible patients per day with the highest CRE risk scores and attempted to approach and consent these individuals. Patients with multiple admissions could be enrolled in the study more than once. Patients absent from their room were revisited twice over two additional days before marking the patient as unavailable. Patients who were deemed too acutely ill to participate by their care team or discharged before initial contact by our research team were not approached for enrollment (Figure 1). Non-English-speaking patients, patients <18 years old, and those on maternity and hospice wards were ineligible. Patients with colostomies were initially excluded, but the protocol was revised approximately one month into the study to include these individuals as they could provide stool samples. The Emory University institutional review board approved this study. Patients or legally authorized representatives provided verbal informed consent prior to participation.

### Sample size determination

Based on prior testing of remnant stool specimens at study hospitals, we estimated that the prevalence of CRE gastrointestinal colonization was 2%. Assuming a CRE prevalence of 2%, we calculated that this study would require a sample size of 322 individuals for estimating the expected proportion with 1% absolute precision and 80% confidence.

### CRE risk prediction score

The CRE risk prediction model was originally developed by the Chicago CDC Prevention Epicenters program and optimized in Georgia using additional data available from the Emory Healthcare EHR.^14,15^ The final model included having a prior infection diagnosis in the past year (based on ICD-10 codes included in an “Epic grouper” utilizing the National Hospital Inpatient Quality Measures (NHQM) Specifications Appendix A, Table 5.09), age, mean length of stay in prior Emory Healthcare acute care hospitalizations, prior admission to an Emory Healthcare intensive care unit in the prior year, Elixhauser comorbidity index score, diabetes mellitus, and antibiotic days of therapy (received during a prior admission at Emory Healthcare) in the past year. This model was built into an automated EHR report that calculates a CRE risk score (probability of having a CRE clinical culture in first 3 hospital days) for each patient (Supplemental Table 1 includes model covariates and parameter estimates).

### Specimen collection and laboratory testing

Consented patients provided either a perianal swab collected by research staff or a self-collected stool sample. Specimens were enriched overnight at 37°C in 5 mL of tryptic soy broth (TSB, BD diagnostics, Franklin Lakes, NJ) and placed on CHROMagar^TM^ extended-spectrum beta-lactamase (ESBL) plates (Hardy Diagnostics, Santa Maria, CA). Colonies from the ESBL plates were identified by MALDI-TOF and automated antimicrobial susceptibility testing was performed on a VitekÒ2 compact instrument using AST-GN74 cards following the manufacturer’s instructions (bioMérieux, Inc Durham, NC). Isolates that were phenotypically resistant to carbapenems were tested for carbapenemase production with the NG-Test® Carba-5R, a rapid lateral flow immunoassay (Hardy Diagnostics). We defined patients as having CRE colonization if their sample identified an Enterobacterales resistant to meropenem (MIC ≥ 4 µg/mL) or ertapenem (MIC ≥ 2 µg/mL).

### Statistical analysis

Descriptive analysis compared demographics and healthcare exposures between enrolled patients with and without CRE colonization, as well as between those who enrolled and those who were originally reviewed but not able to be approached for consent. We also described microbiologic characteristics of the CRE isolates. We used an exact binomial method to estimate the 95% confidence interval (CI) for the CRE screening positivity rate (ie, the percentage of enrolled patients who tested positive for CRE).

To assess model performance, we created a receiver operating characteristic (ROC) curve and calculated the area under the curve (AUC). Using SAS v. 9.4 software (SAS Institute, Cary, NC), we selected five CRE risk scores that spanned the ROC curve to explore cut-points that maximized sensitivity or specificity of using that score as a threshold for CRE testing. We also calculated the positive predictive value (PPV) and negative predictive value (NPV) of each testing threshold. We used the Youden Index (J) which maximizes the sensitivity and specificity of a point on the ROC curve and is often defined as the optimal cut-point for a diagnostic test as the main testing threshold we evaluated. ^17^ Using this threshold we calculated the predictive summary index (PSI) (PSI = PPV + NPV − 1) and number needed to predict (NNP). The NNP is the inverse of the PSI and represents the number of persons who need to be tested to correctly predict (or identify) a diagnosis of the disease (ie, CRE carriage).^19^

### Cost analysis

To estimate the potential costs of implementing a CRE screening program, we first calculated the mean weekly number of patients meeting specified testing thresholds. This was done by dividing the total number of admissions meeting or exceeding the threshold by the total number of sampling days and multiplying by seven. For each screening threshold, the mean number of patients screened per week was combined with the PPV to estimate the expected number patients testing positive for CRE. We used previously published estimates for the costs of laboratory testing and implementation of infection prevention interventions to estimate the total monthly expenditures associated with a CRE screening program at different testing thresholds and then compared this to the estimated cost of managing one healthcare-associated CRE infection (Table 5).^20^ Fixed costs included expenses for specimen collection ($1.00 per swab), culture ($4.09), organism identification and susceptibility testing ($5.70), and phenotypic or molecular confirmation ($37.04). Variable costs include supplies required for contact isolation including disposable gowns, gloves, and other personal protective equipment (PPE), estimated at $58.33 per patient per hospital day.^20^ An additional $15 per screening test was estimated to account for laboratory labor. The average length of stay for CRE colonized patients in our study was 15 days, so we assumed each patient with CRE would require use of PPE for a mean of 13 days after the screening results were available.

**Table 1.** Key characteristics of patients tested for carbapenem-resistant Enterobacterales (CRE) colonization, by colonization status Table 1 long description.

	CRE negative (n = 331)	CRE positive (n = 11)	Total (n = 342)
Age (years), median (IQR)^a^	64 (52–72)	55 (34–65)	64 (52–72)
Male sex, n (%)	168 (51)	5 (46)	173 (51)
Elixhauser comorbidity index score, median (IQR)^a^	10 (4–16)	13 (0–19)	10 (4–16)
Diabetes mellitus, n (%)^a^	91 (28)	2 (18)	93 (27)
Number of prior acute care hospitalizations, median (IQR)^b^	2 (1–4)	4 (1–5)	2 (1–4)
Mean LOS (days) in acute care hospitalizations, median (IQR)^a,b^	7 (3–11)	15 (4–21)	7 (4–12)
Previous admission to ICU, n (%)^a,b^	152 (46)	7 (64)	159 (47)
Prior infection diagnosis per billing codes, n (%)^a,b^	156 (47)	9 (82)	165 (48)
Previous antibiotic DOT, median (IQR)^a,b^	13 (3–33)	34 (3–59)	13 (3–33)
Known prior CRE clinical culture, n (%)	3 (1)	0 (0)	3 (1)
CRE risk score, median % (IQR)	0.04 (0.02–0.09)	0.19 (0.04–0.36)	0.04 (0.02–0.10)

a Variable was used in the model to create the CRE risk prediction score.

b In prior 365 calendar days.

IQR, interquartile range; LOS, length of stay; ICU, intensive care unit; DOT, days of therapy; CRE, carbapenem-resistant Enterobacterales.

**Table 2.** Carbapenem-resistant Enterobacterales automated antibiotic susceptibility and carbapenemase testing Table 2 long description.

Isolate	Species	Ceftriaxone MIC	Meropenem MIC	Ertapenem MIC	Carbapenemase Testing
1	*Enterobacter cloacae* complex	≥64	≤0.25	4	Negative
2	*Klebsiella pneumoniae*	≥64	≥16	≤0.5	KPC
3^a^	*Escherichia coli*	≥64	≥16	≤0.5	KPC
4	*Escherichia coli*	≥64	≥8	≥16	NDM
5^a^	*Escherichia coli*	8	≥16	≤0.5	KPC
6	*Pantoea spp.*	≥64	≤0.25	4	Negative
7	*Klebsiella pneumoniae*	≥64	≥16	≥8	Negative
8	*Klebsiella pneumoniae*	≥64	≥16	≥8	Negative
9	*Klebsiella pneumoniae*	≥64	≥16	≥8	Negative
10	*Enterobacter cloacae* complex	≥64	≤0.25	2	Negative
11	*Enterobacter cloacae* complex	N/A^b^	≥16	2	KPC

a Isolates 3 and 5 represent the same patient in two separate encounters.

b Due to an automatic Vitek suppression rule.

MIC, minimum inhibitory concentration; KPC, *Klebsiella pneumoniae* carbapenemase, NDM, New Delhi metallo-β-lactamase.

**Table 3.** Testing characteristics based on using different CRE risk scores as the threshold for testing Table 3 long description.

CRE risk score (%)	Sensitivity (%)	Specificity (%)	PPV (%)	NPV (%)	Average number of patients meeting this threshold/week^a^
0.01	100	0.6	3.2	100	560
0.07	63.6	57.1	4.7	97.9	58
0.16^b^	54.5	84.3	10.3	98.2	20
0.22	45.5	90.3	13.5	98.0	12
0.36	27.3	96.1	18.8	97.5	5

a Average number of patients at this CRE risk score across all days of testing in our study, multiplied by 7 (for weekly value) and rounded to the nearest integer.

b This threshold value optimizes sensitivity and specificity as determined by the Youden’s Index.

PPV, positive predictive value; NPV, negative predictive value; CRE, carbapenem-resistant Enterobacterales.

**Table 4.** Two by two table depicting carbapenem-resistant Enterobacterales carriage detection among patients tested if we used a testing threshold of ≥0.16% Table 4 long description.

	CRE positive (n = 11)	CRE negative (n = 331)	Total
Above CRE testing threshold (CRE risk score ≥0.16%)	6 (TP)	52 (FP)	58
Below CRE testing threshold (CRE risk score <0.16%)	5 (FN)	279 (TN)	284
Total	11	331	342

TP, true positive; FP, false positive; FN, false negative; TN, true negative; CRE, carbapenem-resistant Enterobacterales.

**Table 5.** Cost breakdown of screening admitted patients at selected carbapenem-resistant Enterobacterales (CRE) risk % prediction thresholds to test patients for CRE carriage Table 5 long description.

		Testing threshold (number of patients that would be screened per month)					
		0.16% threshold (80 patients)		0.22% threshold (48 patients)		0.36% threshold (20 patients)	
Category	Variable	CRE −	CRE +	CRE −	CRE +	CRE −	CRE +
Patients	Patients screened/month	72	8	42	6	16	4
	Total days requiring contact isolation^a^	0	104	0	78	0	52
Fixed costs	Laboratory labor @ $15^b^	$1,080	$120	$630	$90	$240	$60
	Specimen swab @ $5.09^c^	$366	$41	$214	$31	$81	$20
	Organism identification @ $42.74^c^	$0	$342	$0	$256	$0	$171
Variable costs	Per day CI use @ $58.33^c^	$0	$6,066	$0	$4,550	$0	$3,033
Monthly costs	Sum for the month	$1,446	$6,569	$844	$4,927	$321	$3,284
	Sum across CRE status	$8,015		$5,771		$3,605	

a Days in contact isolation is calculated by taking the average length of hospitalization for CRE colonized patients (15 days) and subtracting the first two days from admit date to account for the delay between swabbing and receiving a positive screening result (13 days).

b Estimated labor costs for running lab tests to identify organism.

c Estimates obtained from Maryland CRE modeling study.^20^

CI, contact isolation; CRE, carbapenem-resistant Enterobacterales.

## Results

We sampled patients on 89 days including 10,940 eligible admitted patients. Study staff reviewed the EHR of 1,126 admissions (10%) with the highest CRE risk scores. 853 patients were approached for consent, and 342 (40%) were successfully enrolled in the study (Figure 1). Characteristics of the enrolled patients were similar to those who refused and those who were reviewed but not able to be approached for the study (Supplemental Table 2). We identified CRE in 11 (3.2%, 95% CI: 1.6%–5.7%) of the 342 samples tested. Of note, twenty patients were enrolled in the study more than once (on separate admissions), and two of the positive CRE samples were from the same patient. Patients with CRE were more likely to have had healthcare exposures and underlying illness than patients who tested negative. The median CRE risk score of those with CRE was higher than those who tested negative (0.19% vs 0.04%) (Table 1).

Among the 11 CRE isolates, 4 (36%) were *Klebsiella pneumoniae*, 3 (27%) were *Escherichia coli*, 3 (27%) were *Enterobacter cloacae complex,* and 1 (9%) was a *Pantoea* species. We identified *Klebsiella pneumoniae* carbapenemase (KPC) production in 4 (36%) isolates and New Delhi metallo-β-lactamase (NDM) production in 1 (9%) isolate; we did not identify carbapenemase production in 6 (43%) isolates (Table 2). None of the patients who were identified to have CRE colonization by our testing had a known history of CRE infection. Three of the 11 patients had been placed on contact transmission-based precautions (eg, gowns and gloves required) during their admission for reasons other than CRE (eg, for *Clostridioides difficile* or *Candida auris*).

**Figure 2.**
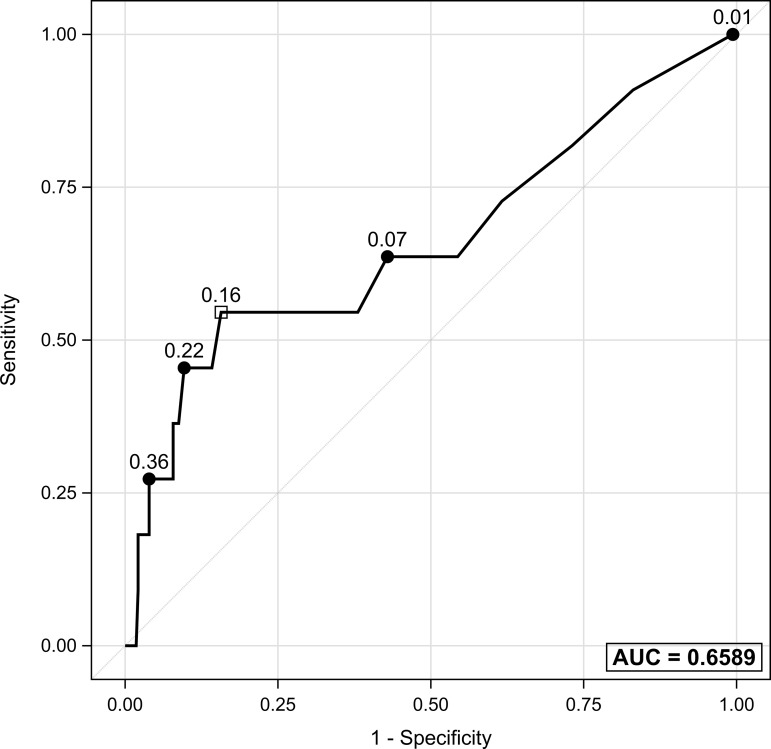
Figure 2 long description. Receiver operating characteristic (ROC) Curve. The ROC curve illustrates the performance of the carbapenem-resistant Enterobacterales risk prediction model. The x-axis represents the false positive rate (1-specificity), and the y-axis represents the true positive rate (sensitivity). The plotted points represent the CRE risk scores that we evaluated as threshold values for testing (see Table 3). The value demarcated by the open square indicates the threshold value that optimized both sensitivity and specificity by the Youden’s Index. AUC, area under the curve.

The AUC of the model incorporated into the EHR report was 0.66 (Figure 2). Table 3 demonstrates testing characteristics for different cut-points or thresholds that could be used for CRE testing. Using the Youden’s Index, the optimal testing threshold was calculated to be a CRE risk score of 0.16%. This testing threshold yielded a 55% sensitivity, 84% specificity, 10% PPV, and a 98% NPV to detect patients with CRE colonization (Table 3). An estimated 20 patients per week at our study hospitals would meet or exceed this threshold for CRE testing on admission (Table 3). Based on the sample of patients we tested, 58 (17%) met this threshold for testing (ie, they had CRE risk score ≥0.16%) and 6 (10%) of those that met the threshold were positive for CRE (Table 4). The NNP using this testing threshold is 12, meaning 12 patients with a CRE risk score ≥0.16% would need to be tested to diagnose 1 patient with CRE carriage.

### Cost analysis

At our selected testing threshold (CRE risk of 0.16%), approximately 80 patients would be screened and 8 patients with CRE identified per month. Applying the cost parameters described, in an average month, the estimated fixed costs for 72 CRE-negative and 8 CRE-positive patients were $1,446 and $503, respectively. Variable costs for PPE among the 8 identified CRE carriers totaled $6,066, assuming each remained hospitalized for a mean of 13 days after screening results were available. In total, the estimated monthly expenditure for the CRE screening program was $8,015 (Table 5). Using $30,484 as the estimated cost of a healthcare-associated infection with CRE, this screening program could be cost-effective if it prevented at least one healthcare-associated CRE infection every three months. Adjusting the testing threshold may influence the cost-effectiveness of a CRE screening program (Table 5).^20^

## Discussion

By prospectively validating and implementing an EHR-based CRE risk prediction model to identify patients for targeted CRE testing on admission in over 300 hospitalized patients, we identified approximately 3% of those tested as CRE carriers. The model demonstrated moderate discriminative performance (AUC of 0.66) under real-world conditions, comparable to that in the original derivation cohort (AUC, 0.73; 95% CI, 0.60–0.87).^15^ Although CRE carriage was rare in our hospitals, limiting testing to patients at highest risk appeared feasible and cost-conscious, especially as the model was programmed into an automated EHR report.

Although this study was performed by research staff, we evaluated the feasibility and practical considerations of implementing a CRE admission screening program, performed by clinical staff, at our study hospitals. We chose a testing “threshold” (ie, all patients who had a model-derived CRE risk score greater than this threshold would be tested) that maximized sensitivity and specificity. At this threshold, approximately 20 admitted patients per week would need to be screened for CRE and 2 would be expected to be CRE carriers. Hospitals could modify the threshold to balance resource use and predictive yield. For example, using a higher cutoff (≥0.36%) may be more practical as it would require screening fewer patients (∼5/week) with a higher PPV (19%), while only sacrificing a small decrease in NPV (98.2% to 97.5%). Tailoring implementation to local CRE prevalence considerations and staffing resources could enhance the efficiency of CRE detection and sustainability of a CRE screening program. Additional implementation considerations include identifying responsibility for running the daily EHR report, conducting the perianal swabs, transporting swabs to the lab, ensuring adequate lab staffing and test turnaround time, integrating results into the EHR, and implementing actions based on screening outcomes. Many of these preanalytical concerns are discussed by Shimasaki et al in their description of implementing targeted CRE admission screening programs in intensive care units.^21^

Active surveillance for CRE in acute care hospitals in the United States is rare, and public health guidance is open to interpretation regarding admission screening.^10,22^ A systematic review of screening strategies to detect carbapenem-resistant gram-negative bacteria found that active surveillance or screening programs are likely effective at decreasing the prevalence of CRE colonization and infection, although the certainty of evidence was low.^23^ In our basic cost-savings analysis, screening for CRE in our study hospitals would potentially be cost-effective, at the testing threshold chosen, if this initiative were able to prevent at least one healthcare-associated CRE case every three months. We did not conduct a formal cost-effectiveness analysis, nor were we able to account for potential cost savings from earlier recognition of patients with CRE or transmission prevention, which would allow for a more complete understanding of the effectiveness of admission screening programs. Larger scale modeling efforts have not consistently shown targeted CRE screening based on admission data to be cost-effective.^20^ A model from the University of Maryland suggested that a registry-based screening approach may be more cost-effective than admission risk model-based triggers.^20^ However, registry-based screening is dependent on having external data sharing agreements which are not available to many healthcare facilities. Additional analyses incorporating transmission dynamics and labor costs are needed to determine the true cost-effectiveness of targeted CRE screening.

Strengths of this study include the prospective, real-time validation of an EHR-integrated CRE prediction model and its evaluation under real-world conditions. Nonetheless, several limitations should be noted. CRE colonization was rare (3.2%), resulting in a low PPV, wide CIs, and a model that performed only moderately well at detecting CRE. For this study, we also defined CRE carriage as any patient with an Enterobacterales isolate phenotypically resistant to meropenem or ertapenem. This differed from the original model creation definition which did not include ertapenem due to limitations in the initial data set. This resulted in some isolates that were only resistant to ertapenem, and these are less likely to harbor carbapenemase genes and may be of lower epidemiologic priority for screening and identification.^24^ Additionally, the study was conducted in a single academic healthcare network, which may limit generalizability. This model may not be adequate in locations where patients commonly seek healthcare from many different healthcare facilities and are unlikely to stay within the same system. Use of a non-random, convenience sample with the need for informed consent likely introduced selection bias but allowed us to enrich for a sample more likely to be colonized with CRE. The AUC curve likely doesn’t capture all patients with a very low risk of CRE, but we still think it is informative to use in determining a potential testing threshold for patients at higher risk for CRE. Additionally, enrollment of patients who were acutely ill, and may be at high risk for CRE, was challenging. However, in our analysis the patients who were not approached due to acuity of illness (n = 37) did not have significantly different CRE risk scores than patients enrolled. We have not assessed the impact of any unintended consequences of infection prevention–based alerts, such as the influence on empiric antibiotic decisions. Finally, our cost analysis was limited by incomplete data on implementation costs and difficulty in estimating the benefit of identifying CRE colonization and the impact this may have on CRE transmission prevention.

In conclusion, our findings indicate that an EHR-based CRE risk prediction tool performs moderately well at identify patients at highest risk for colonization. In settings with low CRE prevalence, screening a targeted, high-risk subset of patients may represent a pragmatic, resource-efficient infection prevention strategy. Future work involves determining how transferrable this model is to other healthcare facilities or regions in the United States. The code for this model can be shared with other facilities using the same EHR, which could allow for streamlined implementation prior to any adjustments or local validation efforts which may include training the model and modifying the coefficients of the covariates.^25^ After implementation, healthcare facilities could adjust testing threshold levels based on local epidemiology and operational capacity.

## Supporting information

10.1017/ice.2026.10488.sm001 Kim et al. supplementary material Kim et al. supplementary material

## Data Availability

The data sets generated and/or analyzed for the current study are to be available in the Emory Dataverse repository (URL: https://dataverse.unc.edu/dataverse/Emory). All personally identifying information (eg, names, addresses, phone numbers, etc.) are absent from the data in compliance with HHS Guidance Regarding Methods for De-identification of Protected Health Information in Accordance with the HIPAA Privacy Rule.

## Supplementary material

The supplementary material for this article can be found at https://doi.org/10.1017/ice.2026.10488.

## Figure 1. Long description

The flowchart begins with 10,940 patients eligible for inclusion, divided between Site A with 6,946 patients and Site B with 3,994 patients. Of these, 1,126 charts are reviewed, with 760 from Site A and 366 from Site B. 274 patients are not approached for consent due to reasons such as patient acuity, unavailability, discharge, limited capacity, or other factors. 852 patients are approached for consent, with 559 from Site A and 293 from Site B. Of these, 342 patients consent and enroll, with 216 from Site A and 126 from Site B. 510 patients refuse consent, with 343 from Site A and 167 from Site B.

Navigate back to Figure 1..

## Table 5. Long description

The table presents a cost breakdown of screening admitted patients for carbapenem-resistant Enterobacterales (CRE) at selected risk prediction thresholds. It includes data for three testing thresholds: 0.16%, 0.22%, and 0.36%, corresponding to 80, 48, and 20 patients respectively. The table is divided into categories: Patients, Fixed Costs, Variable Costs, and Monthly Costs. For each threshold, it shows the number of patients screened per month, total days requiring contact isolation, and various cost components such as laboratory labor, specimen swab, organism identification, and per day contact isolation use. The costs are further broken down into CRE negative and CRE positive cases. Notable trends include higher costs associated with higher thresholds and CRE positive cases requiring more days of contact isolation and higher variable costs.

Navigate back to Table 5..

## Table 1. Long description

The table compares characteristics of patients tested for carbapenem-resistant Enterobacterales (CRE) colonization, divided into CRE negative and CRE positive groups, with a total of 342 patients. It includes data on age, sex, Elixhauser comorbidity index score, diabetes mellitus, number of prior acute care hospitalizations, mean length of stay (LOS) in acute care hospitalizations, previous admission to ICU, prior infection diagnosis per billing codes, previous antibiotic days of therapy (DOT), known prior CRE clinical culture, and CRE risk score. The table has 13 rows and 5 columns, with column headers including Age (years), median (IQR); Male sex, n (percentage); Elixhauser comorbidity index score, median (IQR); Diabetes mellitus, n (percentage); Number of prior acute care hospitalizations, median (IQR); Mean LOS (days) in acute care hospitalizations, median (IQR); Previous admission to ICU, n (percentage); Prior infection diagnosis per billing codes, n (percentage); Previous antibiotic DOT, median (IQR); Known prior CRE clinical culture, n (percentage); CRE risk score, median percentage (IQR). Notable trends include higher median age, Elixhauser comorbidity index score, number of prior acute care hospitalizations, mean LOS in acute care hospitalizations, previous admission to ICU, prior infection diagnosis per billing codes, previous antibiotic DOT, and CRE risk score in CRE positive patients compared to CRE negative patients. Additionally, CRE positive patients have a higher percentage of prior infection diagnosis per billing codes and previous admission to ICU.

Navigate back to Table 1..

## Table 2. Long description

The table presents data on antibiotic susceptibility and carbapenemase testing for 11 isolates of carbapenem-resistant Enterobacterales. It includes columns for isolate number, species, ceftriaxone MIC, meropenem MIC, ertapenem MIC, and carbapenemase testing results. The species listed include Enterobacter cloacae complex, Klebsiella pneumoniae, Escherichia coli, and Pantoea species. The table shows varying levels of resistance to ceftriaxone, meropenem, and ertapenem, with some isolates testing positive for carbapenemase production, specifically KPC and NDM.

Navigate back to Table 2..

## Figure 2. Long description

A line graph illustrates the performance of the carbapenem-resistant Enterobacterales risk prediction model. The x-axis represents the false positive rate (1-specificity), and the y-axis represents the true positive rate (sensitivity). The plotted points represent the CRE risk scores evaluated as threshold values for testing. The value demarcated by the open square indicates the threshold value that optimized both sensitivity and specificity by the Youden’s Index. The area under the curve (AUC) is 0.6589. All values are approximated.

Navigate back to Figure 2..

## Table 3. Long description

The table presents testing characteristics for various CRE risk score thresholds. It includes columns for CRE risk score percentage, sensitivity percentage, specificity percentage, PPV percentage, NPV percentage, and the average number of patients meeting the threshold per week. The rows detail specific thresholds: 0.01 percentage with 100 percentage sensitivity, 0.6 percentage specificity, 3.2 percentage PPV, 100 percentage NPV, and 560 patients per week; 0.07 percentage with 63.6 percentage sensitivity, 57.1 percentage specificity, 4.7 percentage PPV, 97.9 percentage NPV, and 58 patients per week; 0.16 percentage with 54.5 percentage sensitivity, 84.3 percentage specificity, 10.3 percentage PPV, 98.2 percentage NPV, and 20 patients per week; 0.22 percentage with 45.5 percentage sensitivity, 90.3 percentage specificity, 13.5 percentage PPV, 98 percentage NPV, and 12 patients per week; 0.36 percentage with 27.3 percentage sensitivity, 96.1 percentage specificity, 18.8 percentage PPV, 97.5 percentage NPV, and 5 patients per week. The table helps determine the optimal threshold for CRE testing based on sensitivity, specificity, PPV, and NPV.

Navigate back to Table 3..

## Table 4. Long description

The table presents data on carbapenem-resistant Enterobacterales (CRE) carriage detection among patients tested with a threshold of 0.16 percent. It has two rows and four columns. The columns are labeled ‘CRE positive’, ‘CRE negative’, and ‘Total’. The rows are labeled ‘Above CRE testing threshold (CRE risk score greater than or equal to 0.16 percent)’ and ‘Below CRE testing threshold (CRE risk score less than 0.16 percent)’. The table shows that 6 patients above the threshold were CRE positive, while 52 were CRE negative. Below the threshold, 5 patients were CRE positive, and 279 were CRE negative. The total number of patients tested was 342, with 11 CRE positive and 331 CRE negative.

Navigate back to Table 4..
