# Supplementary material for: Prospective validation and implementation of a model to identify patients with carbapenem-resistant Enterobacterales (CRE) carriage on admission to acute care hospitals
Source: Infect Control Hosp Epidemiol. 2026 Jun 16:1–7. Online ahead of print. doi: 10.1017/ice.2026.10488 (PMC13311450; doi:10.1017/ice.2026.10488)
Supplement: Kim et al. supplementary material [file S0899823X26104887sup001.pdf]

Supplemental Table S1. Details of CRE risk prediction model

**Parameter**	**Estimate**
Intercept	-10.7102
Prior infection diagnosis per billing codes[a]	1.058
Previous admission to ICU[a]	0.7964
Age	0.0129
Elixhauser comorbidity index score	0.0338
Mean LOS (days) in acute care hospitalizations[a]	0.0143
Previous antibiotic DOT[a]	0.3348
Diabetes	0.5179

[a]In prior 365 calendar days

Abbreviations: Days of Therapy (DOT); Intensive Care Unit (ICU); Length of Stay (LOS)

Supplemental Table S2. Key characteristics of individuals not approached for enrollment and those who refused enrollment[a]

Characteristic	Patient Acuity (n=37)	Patient Unavailable (n=131)	Patient Discharged (n=74)	LAR Unavailable (n=26)	Other (n=6)	Refused (n=510)
Age (years), median (IQR)[a]	67 (49–75)	63 (49–75)	64 (57–72)	64 (57–72)	69 (31–76)	64 (52–73)
Male sex, n (%)	17 (46)	62 (47)	39 (53)	14 (54)	3 (50)	245 (48)
Elixhauser Score, median (IQR)[a]	11 (5–16)	9 (4–16)	9 (3–17)	7 (0–10)	8 (2–17)	10 (5–16)
Diabetes, n (%)[a]	4 (11)	36 (28)	26 (35)	10 (39)	1 (17)	157 (31)
Number of hospitalizations, median (IQR)[b]	2 (1–4)	2 (1–4)	2 (1–3)	2 (1–4)	4 (2–20)	2 (1–4)
Mean LOS (days) in acute care hospitalizations, median (IQR)[a,b]	5 (2–9)	5 (1–11)	4 (2–8)	6 (3–10)	4 (4–5)	5 (2–10)
Previous admission to ICU, n (%)[a,b]	17 (46)	58 (44)	32 (43)	12 (46)	4 (67)	237 (47)
Prior infection diagnosis per billing codes, n (%)[a,b]	15 (41)	69 (53)	36 (49)	14 (54)	5 (83)	246 (48)
Previous antibiotic DOT, median (IQR)[a,b]	11 (0–20)	9 (0–37)	7 (1–26)	11 (3–23)	19 (11–27)	10 (2–24)
Known prior CRE culture, n (%)	1 (3)	2 (2)	2 (3)	0 (0)	0 (0)	4 (1)
CRE risk prediction score %, median (IQR)	0.05 (0.02–0.09)	0.05 (0.02–0.12)	0.05 (0.02–0.09)	0.07 (0.02–0.12)	0.10 (0.08–0.12)	0.05 (0.02–0.11)

[a]See Figure 1 for more detail on study recruitment and enrollment. The groups of patients here are mutually exclusive.

Variable was used in the model to create the CRE risk prediction score

[b]In prior 365 calendar days

Abbreviations: Legally Authorized Representative (LAR), Interquartile Range (IQR); Length of Stay (LOS); Intensive Care Unit (ICU); Days of Therapy (DOT), Carbapenem-Resistant Enterobacterales (CRE)
